# Primary malignant melanoma of the esophagus successfully treated with nivolumab: a case report

**DOI:** 10.1186/s13256-021-02821-6

**Published:** 2021-05-05

**Authors:** Shingo Ito, Yuji Tachimori, Yuichi Terado, Ryota Sakon, Kazuhiro Narita, Manabu Goto

**Affiliations:** 1 Department of Gastroenterological Surgery, Kawasaki Saiwai Hospital, 31-27 Omiyacho, Saiwai-ku, Kawasaki city, Kanagawa 212-0014 Japan; 2 Department of Pathology, Kawasaki Saiwai Hospital, Kanagawa, Japan

**Keywords:** Malignant melanoma, Nivolumab, Esophagus

## Abstract

**Introduction:**

Primary malignant melanoma of the esophagus is a rare and aggressive disease that tends to have a poor response to chemotherapies. Previous studies have indicated that currently available treatment for primary malignant melanoma of the esophagus is insufficient. Here, we describe a case of recurrent primary malignant melanoma of the esophagus successfully treated with the immune checkpoint inhibitor nivolumab.

**Case presentation:**

An 81-year-old Japanese female presented with a 3-month history of dysphagia. She was medicated for hypertension and sarcoidosis. The patient had no past history of cutaneous, ocular, or other-site melanomas. An esophagoscopy identified a 30-mm giant tumor in the lower esophagus, at a site 30 cm from the incisors. Enhanced computed tomography revealed wall thickening measuring 30 mm in size at the middle-third of the intrathoracic esophagus, with no significant lymph node infiltration or distant metastasis. Esophageal biopsy showed proliferation of large round tumor cells and melanophages. On the basis of these findings, the patient was diagnosed with esophageal malignant melanoma and underwent esophagectomy and lymph node dissection with gastric tube reconstruction. Although the pathological diagnosis was primary malignant melanoma of the esophagus, the patient presented with multiple lymph node and bone metastases 4 months after surgery. Subsequently, treatment with nivolumab 240 mg every 2 weeks was administered as the first-line treatment. Diffusion-weighted imaging with background body signal suppression following eight courses of nivolumab revealed that the multiple lymph node and bone metastases were markedly reduced. The patient received 30 courses of nivolumab and has maintained the partial response. No severe adverse events related to the immunotherapy were recorded.

**Conclusion:**

The current study suggests that nivolumab may be a viable option for patients with metastatic primary malignant melanoma of the esophagus. Additional evidence from future clinical trials and research is necessary to fully validate these findings.

## Background

Primary malignant melanoma of the esophagus (PMME) is an extremely rare disease accounting for 0.1–0.2% of all malignant esophageal tumors, and 0.5% of all noncutaneous melanomas [1, 2]. PMME behaves aggressively and has a poor prognosis and 5-year overall survival (OS) rate of < 5% [3]. PMME patients tend to have a poorer response to chemotherapies than those with other melanomas, and previous studies have indicated that the currently available treatment is insufficient. The clinicopathological characteristics of PMME have been rarely reported, and no comprehensive treatment strategy has been established because of the lack of cases and strong evidence. Recently, immunotherapy has been the preferred choice for unresectable or metastatic melanomas, and as a result, the prognosis of patients with cutaneous metastatic melanoma has improved. Nivolumab is a fully humanized IgG4 programmed death 1 (PD-1) immune checkpoint inhibitor antibody. Compared with dacarbazine, nivolumab treatment has been shown to yield a more favorable survival benefit in previously untreated patients with metastatic melanoma not harboring a BRAF mutation. According to a previous report, a long-term response was achieved with nivolumab in approximately 40% of patients with cutaneous metastatic melanoma [4]. Here, we describe a case of recurrent PMME successfully treated with nivolumab.

## Case presentation

**Fig. 1:**
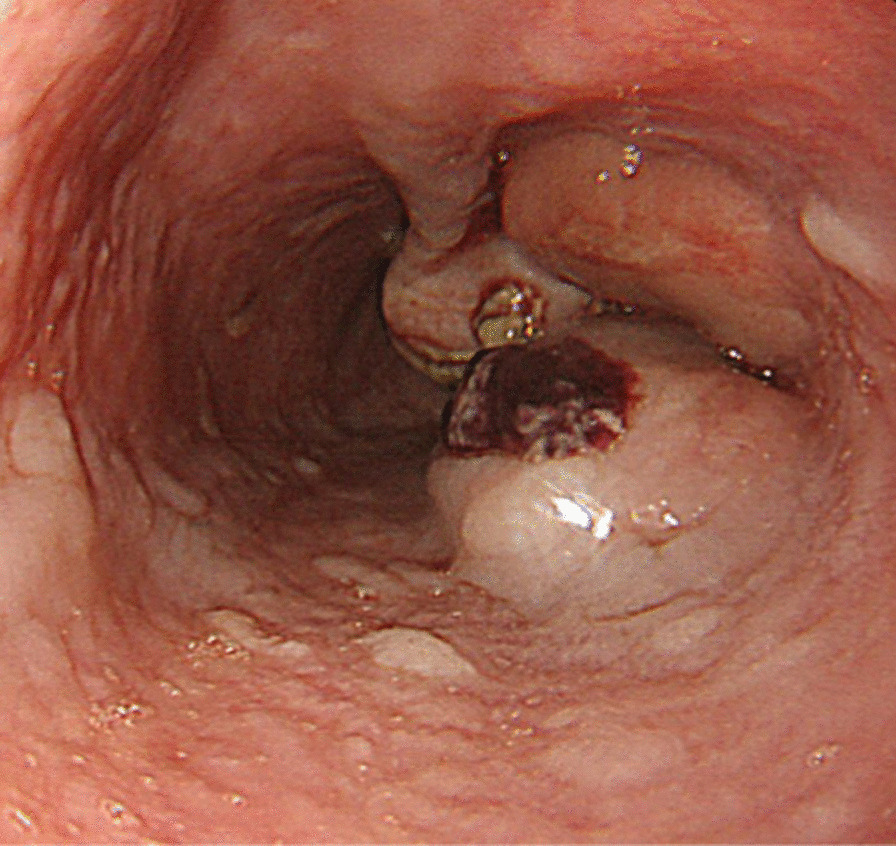
Esophagogastroduodenoscopy revealing a 30-mm giant tumor in the lower esophagus, at a site 30 cm from the incisors

**Fig. 2:**
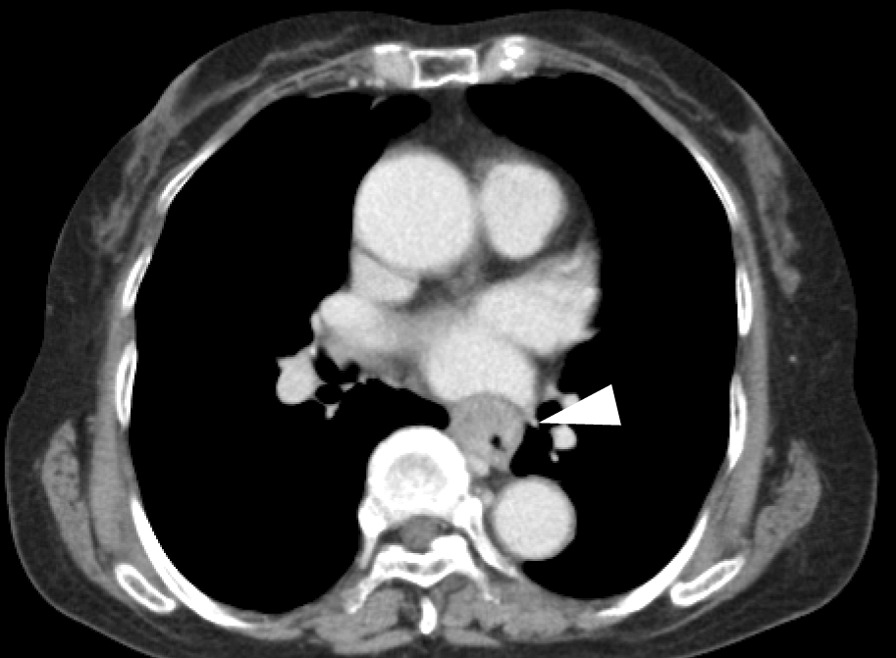
Enhanced computed tomography revealing wall thickening measuring 30 mm in size in the middle-third of the intrathoracic esophagus (arrow head), with no significant lymph node or distant metastasis

**Fig. 3:**
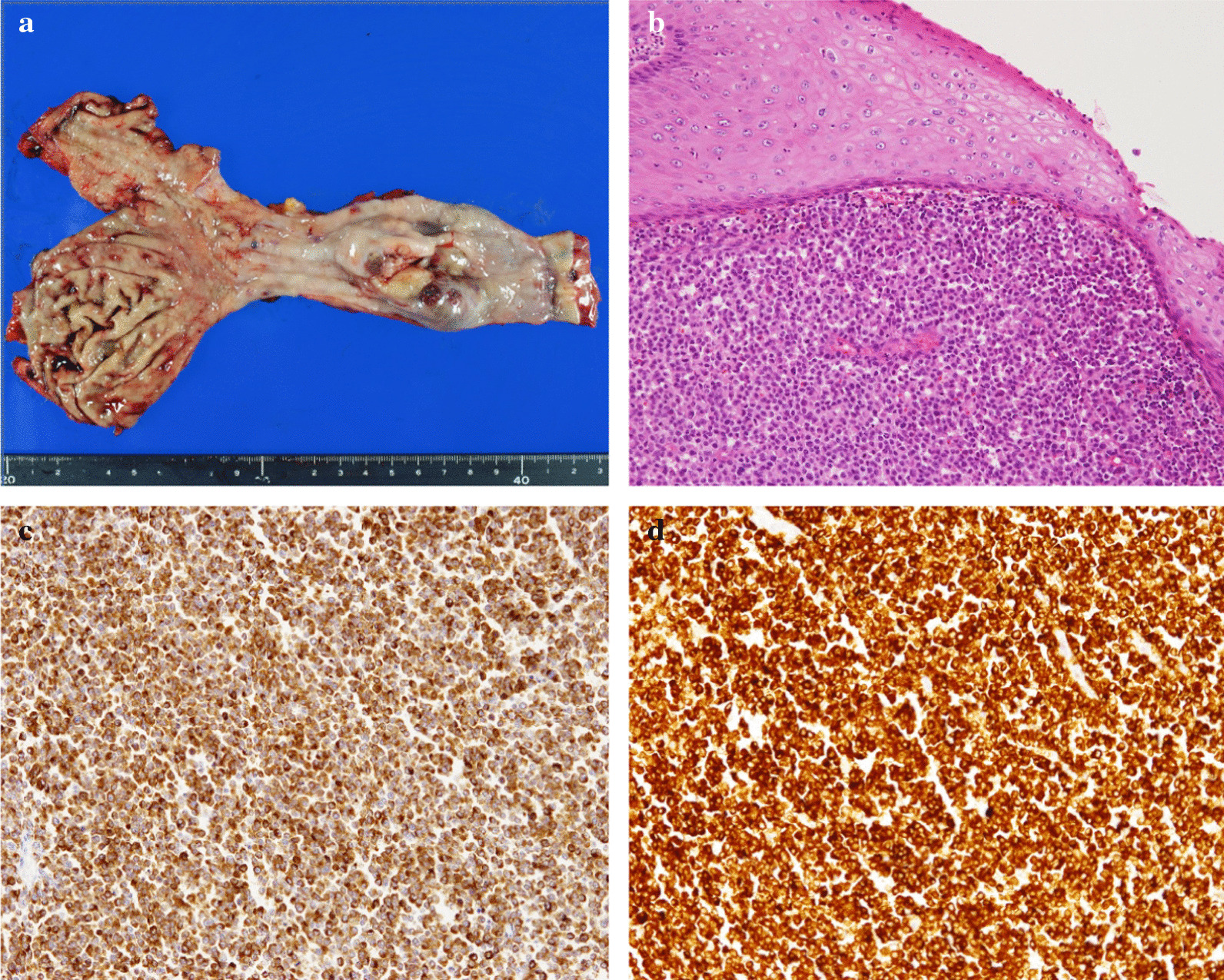
**a** Resected specimen showing an irregular elevated black lesion in the lower esophagus. **b** Hematoxylin–eosin staining of tumor cells. There was solid proliferation, and tumor cells had large round nuclei. Melanin pigmentation was sparse. **c** HMB-45 immunostaining. Tumor cells were diffusely positive. **d** Melan-A immunostaining. Tumor cells were diffusely positive

**Fig. 4:**
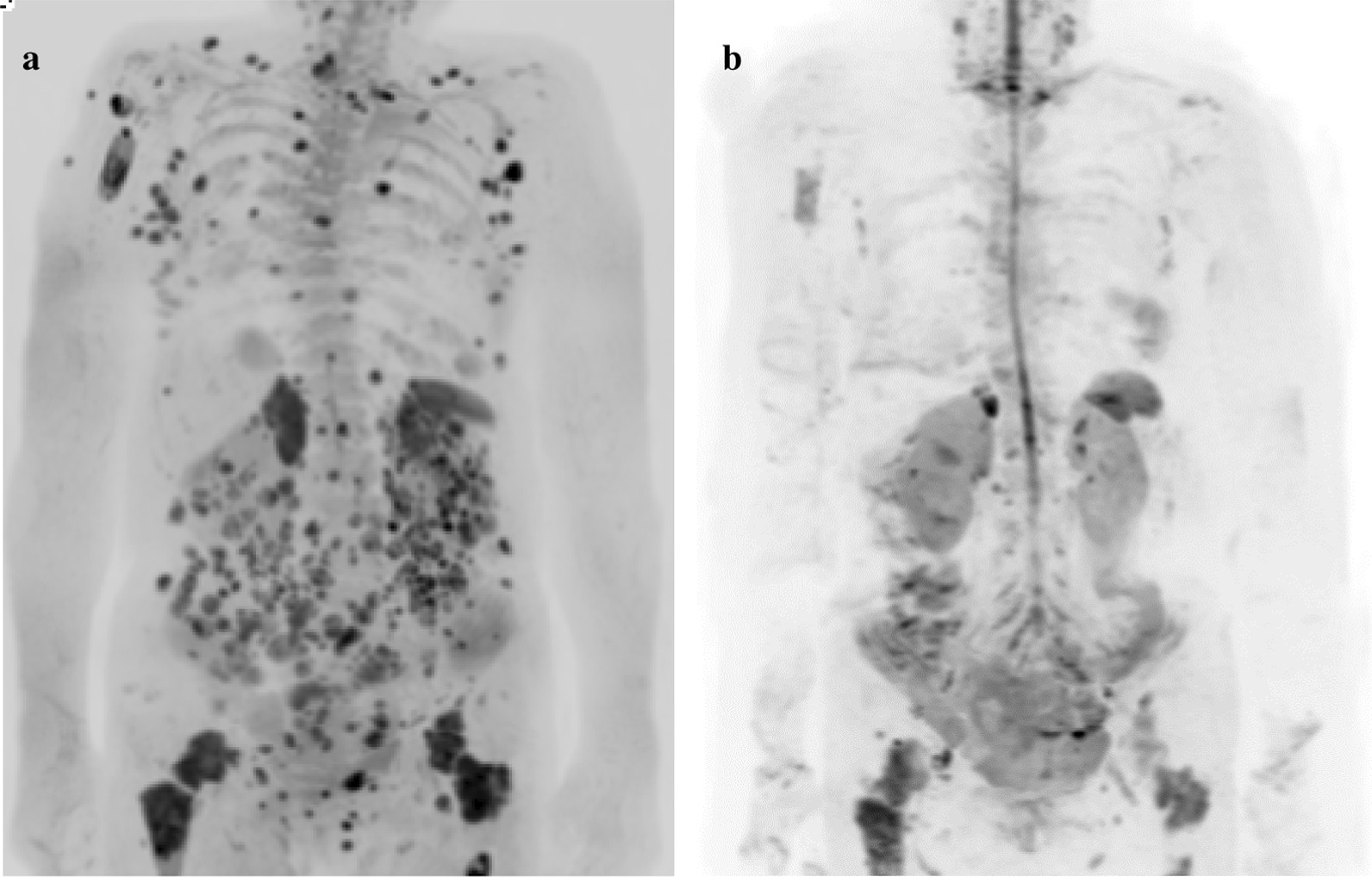
Magnetic resonance imaging (MRI)–diffusion-weighted imaging with background body signal suppression: **a** multiple lymph node and bone metastases before nivolumab treatment; **b** good response after eight cycles of nivolumab

An 81-year-old Japanese female presented with a 3-month history of dysphagia. She was medicated for hypertension and sarcoidosis. She underwent the treatment with an antihypertensive drug. There was no family or medical history of other-site melanomas. Esophagoscopy showed a 30-mm white ulcerating tumor in the lower esophagus, at a site 30 cm from the incisors (Fig. 1). Pathological examination showed an atypical short spindle to polygonal cells, which was positive for human melanoma black (HMB)-45, and melan-A, but negative for anticytokeratins (CK7 and CK20) on immunohistochemical analysis. Enhanced computed tomography revealed wall thickening measuring 30 mm in size in the lower intrathoracic esophagus, with no significant lymph node or distant metastasis (Fig. 2). On the basis of these findings, we diagnosed PMME, and the patient underwent subtotal esophagectomy with two-field lymph node dissection with gastric construction. Macroscopically, the tumor was an irregular elevated white and black mass 60 × 35 mm in size in the lower esophagus (Fig. 3a). A microscopic examination demonstrated that the tumor was located in the submucosal lesion and that there was solid proliferation of eosinophilic tumor cells without tubular or papillary structures (Fig. 3b). The tumor cells had large round nuclei, and melanin pigments were found in the cytoplasm of tumor cells. Positive immunohistochemical staining for HMB45 and melan-A was observed in tumor cells as with the biopsy specimen (Fig. 3c and d). Based on these morphological features and the immunohistochemical findings, the tumor was diagnosed as PMME with T3 invasion, lymph node negative (0/27), and the final disease stage was classified as pT3N0M0 stage III (UICC, eighth edition). The patient refused postoperative adjuvant treatment, and 4 months after surgery, whole-body lymph node and bone metastases appeared. Magnetic resonance imaging (MRI)–diffusion-weighted imaging with background body signal suppression (DWIBS) showed whole-body lymph node swelling and bone metastases (Fig. 4a). As the patient had a nonmutated BRAF gene, she was started on intravenous administration of nivolumab (240 mg, every 2 weeks) according to the National Comprehensive Cancer Network (NCCN) malignant melanoma treatment guideline [5]. After eight cycles, the lymph node and bone metastases were dramatically reduced. Following eight courses of nivolumab, MRI-DWIBS revealed that the multiple lymph node and bone metastases were markedly reduced (Fig. 4b). Blood tests showed transient elevation of serum lactate dehydrogenase (LDH) to 4000 IU/L before the initiation of nivolumab treatment. However, the serum LDH levels returned to a normal range 3 months after the treatment. The patient received 30 courses of nivolumab for 15 months after recurrence. During the treatment, Grade 1 fatigue was the only adverse event observed, and she had maintained the partial response for 15 months. However, bilateral adrenal metastases occurred. Nivolumab therapy and all other treatment were then stopped on the patient’s request. She died about 27 months after recurrence. No severe immunotherapy-related adverse events (irAE) were recorded.

## Discussion

Here, we report a case of recurrent PMME with lymph node and bone metastases successfully treated with nivolumab. After the treatment, the lymph node and bone metastases showed a dramatic decrease, and no irAE were observed during the treatment. The patient received nivolumab for 16 months and showed no further signs of clinical disease progression. The incidence of malignant melanoma has increased over the past few decades, and approximately 132,000 people develop malignant melanoma each year worldwide [5]. Almost all malignant melanoma cases arise from the skin, and it is reported that only 1% of melanomas arise from the mucosa (head and neck, eyes, and genitourinary and alimentary tracts) [4]. Although mucosal melanomas generally carry a worse prognosis than those arising from cutaneous sites, no intrinsic risk factors and specific treatment options have been established. Furthermore, there is no evidence of a difference in sensitivity between skin and mucosal melanomas to nivolumab therapy. Xuan Wang *et al.* [6] reported that, in 76 PMME patients, PMME occurred more commonly in men, with a male-to-female ratio of 2.17:1. The majority of PMME patients are symptomatic on diagnosis, dysphagia being the most common major symptom of PMME, as was observed in the current case. Concerning the locations of PMME tumors, 92.1% were in the middle and lower portions of the esophagus, while half of the tumors invaded the muscularis propria or further. Consistent with the results of previous studies, the incidence of periesophageal lymph node metastasis did not correlate with the depth of tumor invasion. Surgical resection is the most common treatment, with 77.6% patients undergoing subtotal esophagectomy or esophagogastrostomy with lymph node dissection. Despite complete excision, recurrence occurred in 89.7% of the patients. In addition, the interval between primary surgery and recurrence was only 4.5 months. The risk of recurrence is extremely high after an initial staging operation, which likely reflects the aggressive characteristics of PMME and the important role of adjuvant therapy. Indeed, adjuvant therapy has been shown to increase recurrence-free survival (RFS) and to have varying effects on OS in patients with cutaneous melanoma [7]. A previous trial suggested that temozolomide (TMZ)-based adjuvant chemotherapy can improve both RFS and OS in patients with mucosal melanoma [8]. However, because of its rarity, optimal adjuvant therapies for PMME have not yet been established. Postoperative adjuvant chemotherapy may be considered for patients with PMME because it significantly improves RFS. However, even with adjuvant chemotherapy, the RFS is still much lower than in other subtypes of mucosal melanoma [8]. A phase 3 randomized trial suggested that adjuvant therapy with ipilimumab was a treatment for stage III melanoma based on a significantly prolonged RFS [9]. In addition, CheckMate 238 clinical trials showed that, among patients undergoing resection of stage IIIB, IIIC, or IV melanoma, adjuvant therapy with nivolumab resulted in a significantly longer RFS and a lower rate of grade 3 or 4 adverse events than adjuvant therapy with ipilimumab [10]. Moreover, nivolumab may be effective for patients undergoing resection of stage III or IV PMME as adjuvant therapy.

The role of systematic therapy for metastatic or unresectable PMME remains unclear. The first-line systemic therapy for melanoma is immunotherapy such as nivolumab, ipilimumab, and pembrolizumab according to the NCCN guidelines. In previous studies, traditional cytotoxic chemotherapies have displayed very little efficacy against advanced-stage PMME. The overall response rate of chemotherapy in a previous cohort study was only 10.9%, with short progression-free survival (PFS) of just 3 months [10]. Other studies have also shown unsatisfactory results of chemotherapy. Indeed, Weiner *et al.* [11] reported an OS of patients undergoing chemotherapy of only 7.7 months, and a 3-year OS of 0 in eight patients. The treatment options for patients with metastatic melanoma have improved dramatically in the past 5 years with the development of targeted therapies and immunotherapies. The immune checkpoint inhibitors nivolumab and ipilimumab represent novel treatment strategies for malignant melanoma. These drugs have been reported to demonstrate a substantial clinical benefit for patients with metastatic melanoma, with objective response rates of 31.0–40.0% [12]. A number of previous case reports suggested that the usefulness of immunotherapy with nivolumab for PMME may be comparable to melanoma of other organs [5, 13]. Patients with metastases at the time of diagnosis had a median survival rate of 15.8 months, whereas those who developed metastases later or had unresected stage III disease had an average survival rate of 22.8 months from the date of first diagnosis, and the median OS from the first diagnosis was 18.5 months.

## Conclusion

PD-1 inhibitors may represent a promising option for patients with advanced PMMEs. More evidence is needed in future clinical research to further validate their role.

## Data Availability

No additional dataset was used for creation of this manuscript. All information was available from standard documentation in the patient’s electronic medical record.
